# Design of Hierarchical Structures for Synchronized Deformations

**DOI:** 10.1038/srep41183

**Published:** 2017-01-24

**Authors:** Hamed Seifi, Anooshe Rezaee Javan, Arash Ghaedizadeh, Jianhu Shen, Shanqing Xu, Yi Min Xie

**Affiliations:** 1 Centre for Innovative Structures and Materials, School of Engineering, RMIT University, Melbourne, Victoria 3001, Australia; 2 XIE Archi-Structure Design (Shanghai) Co., Ltd., Shanghai 200437, China

## Abstract

In this paper we propose a general method for creating a new type of hierarchical structures at any level in both 2D and 3D. A simple rule based on a rotate-and-mirror procedure is introduced to achieve multi-level hierarchies. These new hierarchical structures have remarkably few degrees of freedom compared to existing designs by other methods. More importantly, these structures exhibit synchronized motions during opening or closure, resulting in uniform and easily-controllable deformations. Furthermore, a simple analytical formula is found which can be used to avoid collision of units of the structure during the closing process. The novel design concept is verified by mathematical analyses, computational simulations and physical experiments.

Hierarchical structure is one of the omnipresent natural forms in biological systems^1^,^2^. The structural hierarchy is a major feature determining the bulk properties of natural materials^1^,^2^. Inspired by this, man-made hierarchical materials have been extensively developed in the past decades^3^,^4^,^5^,^6^,^7^,^8^,^9^. More recently, there has been increasing interest in creating materials whose mechanical properties are largely determined by their cellular architecture rather than their chemical composition, i.e., the so-called metamaterials^10^,^11^,^12^,^13^,^14^,^15^,^16^,^17^,^18^,^19^,^20^,^21^. Furthermore, metamaterials that are hierarchically constructed have been reported to exhibit exceptional mechanical properties such as high strength, enhanced toughness and reversible extensibility^6^,^9^,^22^,^23^,^24^,^25^,^26^,^27^,^28^. Among the metamaterials, auxetic materials, which exhibit negative Poisson’s ratio (NPR), have been studied extensively^6^,^10^,^11^,^28^,^29^,^30^. To design auxetic metamaterials with tuneable mechanical properties, the mechanism of “rotating rigid units” is one of the most frequently used principles^8^,^9^,^10^,^11^,^12^,^28^. Recently, Cho *et al*.^7^ proposed a simple but ingenious fractal cut method to develop shape-programmable and hierarchical auxetic metamaterials. In their work, the degrees of freedom (DOF) of hierarchical structures would increase greatly with increasing hierarchy levels, which allowed the maximum flexibility in achieving different shapes. A similar concept was proposed by Gatt *et al*.^28^ for designing novel stents and skin grafting patterns using hierarchical auxetics. The concept of hierarchical cut based on rotating rigid units aims to create tuneable metamaterials with exceptional mechanical properties such as extreme expandability and conformability, which shows promising applications in a wide range including stretchable electronics and photonics^28^,^31^, conformable electronic skin^32^, skin graft and biomedical devices^33^. For such applications, higher degrees of freedom are beneficial for shape flexibility. On the other hand, designing hierarchical structures with lower degrees of freedom is also important but has not been extensively studied. Such hierarchical structures could be used as retractable and deployable devices and structures both on macro- and micro-scales in a broad range of industries. However, it is rather complex and difficult to control the deformations of metamaterials if the degrees of freedom are large. Numerous constraints or forces would need to be applied simultaneously, which makes practical applications of such metamaterials extremely difficult when the level of hierarchy is high.

In this study we propose a new approach to create hierarchical metamaterials based on the principle of rotating rigid units. Starting from a proper arrangement of the constructing rigid units and by using the proposed rotate-and-mirror method, we are able to create hierarchical metamaterials with few degrees of freedom. More importantly, our new design concept gives rise to synchronized motions of unit cells of the structure, resulting in controllable uniform deformations. An analytical solution is found for avoiding collision of units during the closure process. The same approach can also be used to create 3D hierarchical metamaterials with synchronized deformations and auxetic properties. We demonstrate our design concept through numerical simulations, mathematical analyses and physical experiments.

## Results

### Rotating rigid units and constructing assemblies

**Figure 1:**
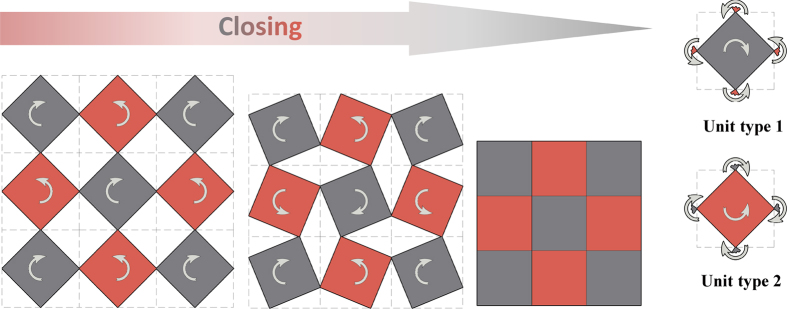
Closing procedure of a typical level-1 structure made of two types of square rotating rigid units, from completely open state to fully closed state. Type 1 unit rotates clockwise and type 2 rotates counter-clockwise, opposite to the rotation directions of adjacent units.

We assume that the base material is undeformable, i.e., rigid, and the connections behave like frictionless rotational hinges, similar to the assumptions made by Cho *et al*.^7^. Thus the deformation of the structure is governed by the rotation of the units rather than the deformation of the base material itself. For simplicity, square-shaped rigid units are used to construct hierarchical structures. Figure 1 shows the level-1 hierarchy. This structure has only one degree of freedom (*F* = 1), meaning the rotation of a single unit will bring synergetic rotation of the entire structure. The closure or opening of a hierarchical structure is the direct result of the rotations of two types of base unit cells, defined by their rotational directions as illustrated in Fig. 1. Type 1 cells rotate clockwise and type 2 cells rotate counter-clockwise. The construction of the level-1 structure is through mirroring a rotated base unit cell about vertical and horizontal lines parallel to the two axes of the original Cartesian coordinate system, which pass through the vertices of the unit. Thus, the rotational direction of this unit is opposite to those of its adjacent units.

**Figure 2:**
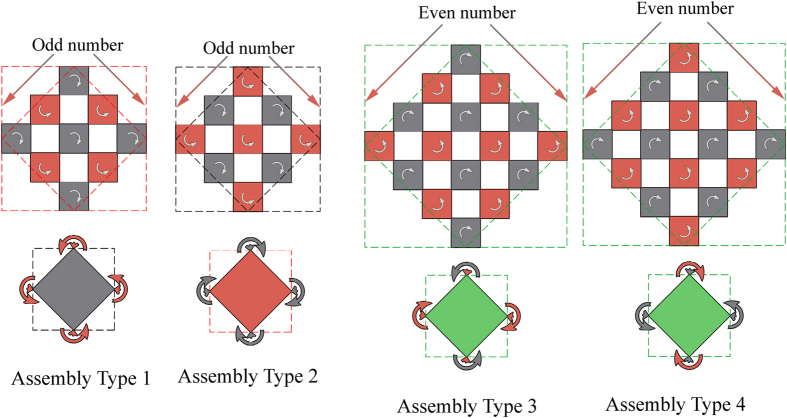
Rotating assemblies for constructing higher level hierarchies. Assembly types 1 and 2 have equal- and odd-numbered units along all edges (to be favoured) while assembly types 3 and 4 have equal- and even-numbered units (to be avoided). The colour arrows illustrate the rotational directions of the adjacent units.

The level-1 structure shown in Fig. 1 could be treated as an assembly and used to construct higher level hierarchies, which has three unit cells along each of its four edges. Actually, there are two types of assemblies could be arranged for level-1 structure as illustrated in Fig. 2, namely type 1 or type 2, depending on their overall rotational directions. Due to geometric constraints, the rotational motion of a rigid unit at a vertex would be transferred to the other three vertices, leading to synchronized rotations of all the four vertices. The remaining units along the four edges would rotate in the opposite direction. Therefore, these two types of assemblies could be treated as equivalent rotating units, similar to the two types of rigid units. It should be noted that, these two types of assemblies have the same odd-numbered rigid units. The numbers of the rigid units along two neighboring edges could also be unequal, creating rectangular assemblies instead of square ones in this work. However, if the number is even, e.g., types 3 and 4 in Fig. 2, the rotational direction of any unit at vertex would rotate in the opposite direction owing to the geometric constraints. As a result, there would be no synchronized rotation for the four vertex units and they cannot be treated as equivalent units. Therefore, to effectively create assemblies with synchronized rotations at all vertices, the number of rotating rigid units along each edge of the assembly should be odd.

### Construction of planar hierarchical structures

**Figure 3:**
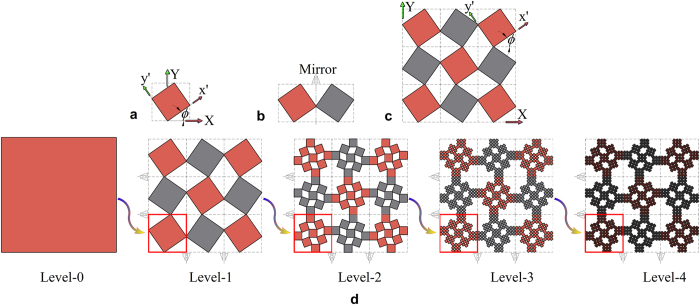
Procedure of building higher level hierarchies through the rotate-and-mirror method. (**a**) rotate a unit or an assembly, (**b**) mirror the unit or assembly, (**c**) put an array of odd-numbered units/assemblies along each edge, and (**d**) a demonstration of building a level-4 hierarchical structure.

Here we propose a general and consistent procedure for constructing hierarchical structures at any hierarchical level, from one level to the next. The construction of the level-(*i* + 1) hierarchical structure is achieved through a rotate-and-mirror procedure from the level-*i* assembly. Firstly, a level-*i* assembly is rotated from its original *X*-*Y* coordinate to the new coordinate *x′*-*y′* by an angle of *ϕ* (0 < *ϕ* ≤ 90°), as shown in Fig. 3a. The rotation procedure can be quantified by using transformation matrix **T** on odd levels and **T**^**−1**^ on even levels (see Supplementary Note 1), i.e., rotating the assembly from odd levels clockwise and rotating the assembly (or rigid unit) from even levels counter-clockwise. This simple rule guarantees that the assemblies are joined together by connecting adjacent units at the edges of the assemblies by *two* vertices. This is fundamentally different from the single vertex connection between neighboring rigid units in all hierarchical structures created by Cho *et al*.^7^. After the aforementioned rotation, a series of mirroring operations are performed to construct the level-(*i* + 1) hierarchy (Fig. 3b,c). Through such operations, odd-numbered units can be assigned to the hierarchical structure, which can be used as the base assembly to construct a higher level structure. The complete process of creating a level-4 hierarchical structure is illustrated in Fig. 3d and more details are given in Supplementary Note 1 and Supplementary Fig. 1.

### Degrees of freedom

The number of degrees of freedom of a hierarchical structure is of critical importance because it determines the deformation mechanisms. For practical applications, smaller degrees of freedom are often desirable so that the deformation of the structure could be controlled with fewer driving forces or independent motors. The increase in the hierarchical level usually results in an enormous increase in the degrees of freedom. However, we have discovered that the hierarchical structures constructed by using our rotate-and-mirror method exhibit remarkably less degrees of freedom than similar designs created by fractal cut method.

**Figure 4:**
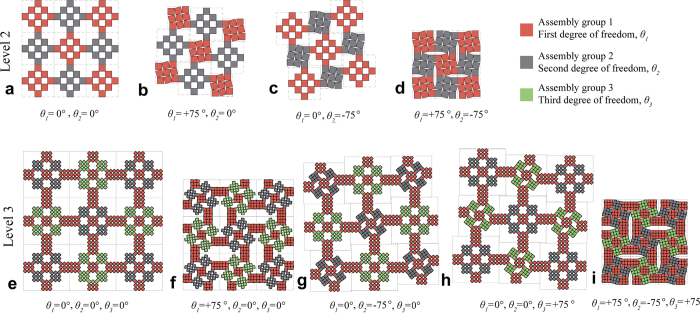
Degrees of freedom for level-2 and level-3 hierarchical structures, *F*_*2*_ = 2 and *F*_*3*_ = 3.

For a hierarchical structure, we may define the angle *θ* to rotate a type 2 (or type 1) rigid unit from its completely open state to the state of construction (positive in clockwise rotation), which is used to characterize the degree of freedom (see Supplementary Note 2). The level-0 structure, which consists of a single rigid square unit, has no independent variable for deformation (i.e., zero degree of freedom, *F*_*0*_ = 0). The level-1 structure has one angle, *θ*, governing the deformation of the entire structure and therefore its degree of freedom is one, i.e., *F*_*1*_ = 1. Our level-2 and level-3 hierarchical structures have two and three degrees of freedom, *F*_*2*_ = 2 and *F*_*3*_ = 3, respectively, as illustrated in Fig. 4. We can also verify that, for level-4 and level-5 hierarchical structures, *F*_*4*_ = 4 and *F*_*5*_ = 5. Therefore, we believe that the degrees of freedom of a hierarchical structure constructed by our rotate-and-mirror method can be simply expressed as *F*_*N*_ = *N*, where *N* is the hierarchical level. The mathematical derivation of the degrees of freedom for hierarchical structures up to level five is given in Supplementary Note 2 and Supplementary Figs 2–6.

**Figure 5:**
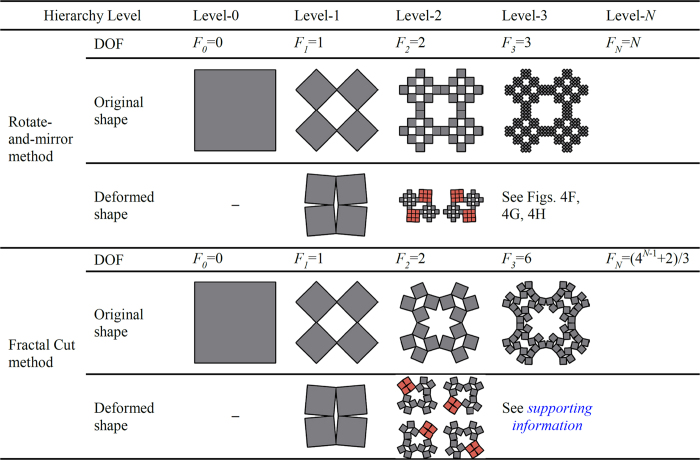
Comparison of degrees of freedom for two types of hierarchical structures constructed by the rotate-and-mirror method and the fractal cut method (7).

In Fig. 5 we compare the hierarchical structures created by our rotate-and-mirror method with designs obtained from the fractal cut method. For example, the level-4 hierarchical structure from our method has *F*_*4*_ = 4, while the level-4 design from the fractal cut has *F*_*4*_ = 22 according to the equation *F*_*N*_ = (4^*N*−1^ + 2)/3 given by Cho *et al*.^7^. Actually, we have used Working Model software to simulate the deformation of the fractal cut designs in the case of unconstrained rotational movement and found that they may have even more degrees of freedom than the reported numbers if the Grubler’s equation was applied (see Supplementary Note 3, Supplementary Figs 7 and 8), which would be beneficial to the expanded applications of fractal cut method in shape engineering. For example, the level-2 structure from the fractal cut method could have more than two degrees of freedom. After extensive numerical simulations, we have found that the degrees of freedom of the fractal cut designs are dependent on the number of assemblies along each edge (see Supplementary Fig. 7), i.e., the degrees of freedom could vary significantly on a given level of hierarchy. In contrast, the degrees of freedom of the hierarchical structures created by our rotate-and-mirror method are independent of the number of assemblies along the edge. Therefore, the present concept of creating hierarchical structures is complementary to the fractal cut method.

In the rotate-and-mirror method, rigid units or assemblies of the same DOF group will rotate synchronously without interfering with other units or assemblies of different DOF groups, which results in uniform deformation in certain regions or the whole structure. Due to the significantly reduced number of degrees of freedom, a synchronized motion and a uniform deformation of a complex high level hierarchical structure can be easily achieved by controlling the rotations of very few base rigid units. Such a feature is of significant importance for the control and operation of retractable roofs and deployable antennae with hierarchical patterns.

### Computational and experimental verifications

**Figure 6:**
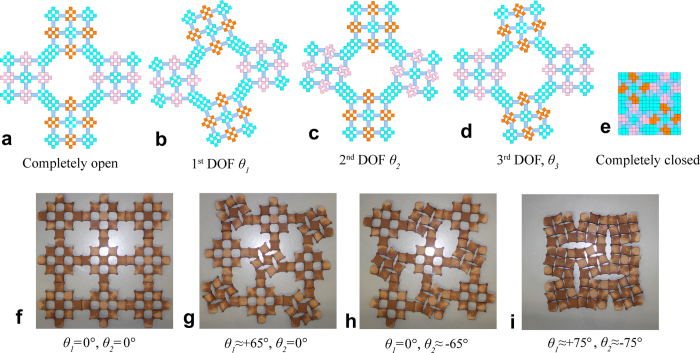
Numerical and experimental verifications of the new hierarchical structures. (**a**) to (**e**) motion simulation of a level-3 hierarchical structure showing three independent degrees of freedom, (**f**) to (**i**) experimental verification of a level-2 hierarchical structure showing two degrees of freedom.

To verify the effectiveness of the proposed design approach and validate the correctness of the mathematical derivation of the degrees of freedom, we perform computational simulations using motion simulation software, Working Model. The motions of hierarchical structures up to three levels constructed by the rotate-and-mirror method are systematically examined. The rotating units are set to be rigid and the vertices are pin jointed. On each level, all degrees of freedom are initially constrained. Then one degree of freedom is released at each step. The results from the motion simulations confirm that the degrees of freedom of the new hierarchical structures are equal to the levels of hierarchy, i.e., *F*_*1*_ = 1, *F*_*2*_ = 2 and *F*_*3*_ = 3. Figure 6a–e shows the deformation patterns of a typical level-3 hierarchical structure. A level-2 structure was also simulated in the same procedure, as shown in Fig. 4a–d.

We also carry out conceptual verification by a series of simple experiments. Using laser cutting, we are able to make square rigid wooden units of the same size, with tiny holes on four vertices. The units are connected together using metal pins. The same rotational loading conditions as in the motion simulations are applied to level-2 hierarchical structure and the resulting deformation patterns are shown in Fig. 6f–i. The results are highly consistent with those from the simulations. A video of one of the experiments is given in Supplementary Video 6.

The computational simulations and experimental observations prove that rigid units or assemblies of the same type will rotate simultaneously in the same manner during the opening or closure of the hierarchical structure, leading to a synchronized deformation. However, collision between neighboring units may occur in structures with three or higher levels of hierarchy. Such a collision would prevent the hierarchical structure from a full closure, which would severely limit its practical applications, e.g., as a retractable roof. To overcome this limitation, we have derived a mathematical formula for the condition that must be satisfied by the rotational angles in order to prevent collision in the level-3 hierarchical structure, which is


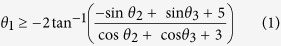


with this formula, a complete closure of the level-3 structure can be achieved by selecting proper rotation sequence among the three degrees of freedom, without any collision between units of different assemblies. We have also applied the same analysis to the level-2 hierarchical structures and found that they would never collide during the closure process Details of the derivation of the above formula and a series of videos demonstrating the validity of the collision condition are provided in Supplementary Note 3, Supplementary Figs 9 to 11, Supplementary Videos 4 and 5.

## Discussion

The rotate-and-mirror method proposed in this paper provides a general method to design hierarchical structures of any level. It is capable of creating hierarchical structures with significantly reduced degrees of freedom, resulting in a high level of controllability. Most importantly, each group of the rotating rigid units or assemblies move synchronously during the opening or closure of the entire structure, leading to predictable and programmable shape transformation. This is of critical importance for applications of hierarchical structures in deployable or retractable systems. A notable application of the proposed method is in designing 2D or 3D auxetic structures due to the significantly reduced area or volume in the process of shape transformation, as illustrated in the experimental realization of the level-2 structure in Fig. 6f–i.

Furthermore we may put different numbers of square units in each type of rotating unit/assembly. For example, in Fig. 3d, the two types of rotating assemblies of the level-2 structure could contain different numbers of square units, resulting in a very different pattern after full closure. Besides, the rigid unit could be in different shapes than square to generate a variety of patterns for different applications. The present method can also be easily extended to constructing 3D hierarchical structures (see Supplementary Note 5, Supplementary Table 1), albeit with more degrees of freedom.

In summary, we propose in this paper a general method for creating a new type of hierarchical structures at any level in both 2D and 3D by using a rotate-and-mirror method. The hierarchical structures designed by this method have remarkably few degrees of freedom equal to their hierarchical levels. The significantly reduced degrees of freedom are caused by the synchronized motion of the hierarchical structure in the opening or closing process, which makes the deformation uniform and easily-controllable. To achieve the desired deformation of the proposed hierarchical structure without collision, a theoretical condition has been derived. Our novel design concept has been verified by mathematical analyses, computational simulations and physical experiments.

## Methods

### Computational simulations

We apply Working Model 2D (version 9.0) in this study to simulate the deformation of 2D hierarchical structures of various levels. Square rigid units are used in constructing hierarchies via pin joints at vertices of neighboring units such that the rigid units can be rotated freely. The gravity of the units in this work is ignored for simplicity. In simulation, we fix a specific unit and apply motors sequentially or simultaneously on vertices of the units belonging to different types of assemblies to mimic their rotational movements. When rotating a selected unit, a displacement constraint is defined on two vertices of the two neighboring units in a different assembly group from the rotated one (see Supplementary Fig. 9), i.e., the so-called “rod” constraint in Working Model. Thus, when one unit of a certain assembly type is restrained (no rotation), the deformation of the remain units of the same assembly type and units of other assembly types can be observed. In this way, we can examine the degrees of freedom of hierarchical structures of different levels and see clearly the deformation mechanisms of the structure. Detailed examples are given in Supplementary Note 3, Supplementary Fig. 9 and Supplementary Videos 1 and 3.

### Fabrication process

Square wooden units of 20 × 20 × 3 mm^3^ are fabricated through laser cutting with holes made in the four vertices for pin-connections. A conceptual level-2 hierarchical structure is then assembled by using pin-connections. In the experiments, the two types of rigid units are rotated individually and sequentially, demonstrating synchronized motion of the same unit type and the degree of freedom for this structure, *F*_*2*_ = 2. Supplementary Video 6 shows the deformation process of the conceptual structure.

## Additional Information

**How to cite this article**: Seifi, H. *et al*. Design of Hierarchical Structures for Synchronized Deformations. *Sci. Rep.* **7**, 41183; doi: 10.1038/srep41183 (2017).

**Publisher's note:** Springer Nature remains neutral with regard to jurisdictional claims in published maps and institutional affiliations.

## Supplementary Material

Supplementary Information

Supplementary Video 1

Supplementary Video 2

Supplementary Video 3

Supplementary Video 4

Supplementary Video 5

Supplementary Video 6
