# Supplementary material for: Design of Hierarchical Structures for Synchronized Deformations
Source: Sci Rep. 2017 Jan 24;7:41183. doi: 10.1038/srep41183 (PMC5259711; doi:10.1038/srep41183)
Supplement: Supplementary Information [file srep41183-s1.pdf]

Supplementary Information

Design of Hierarchical Structures for Synchronized Deformations

Hamed Seifi[1], Anooshe Rezaee Javan[1], Arash Ghaedizadeh[1], Jianhu Shen[1], Shanqing Xu[1], and Yi Min Xie[1,2,*]

[1]Centre for Innovative Structures and Materials, School of Engineering, RMIT University, Melbourne, Victoria 3001, Australia. [2]XIE Archi-Structure Design (Shanghai) Co., Ltd., Shanghai 200437, China.

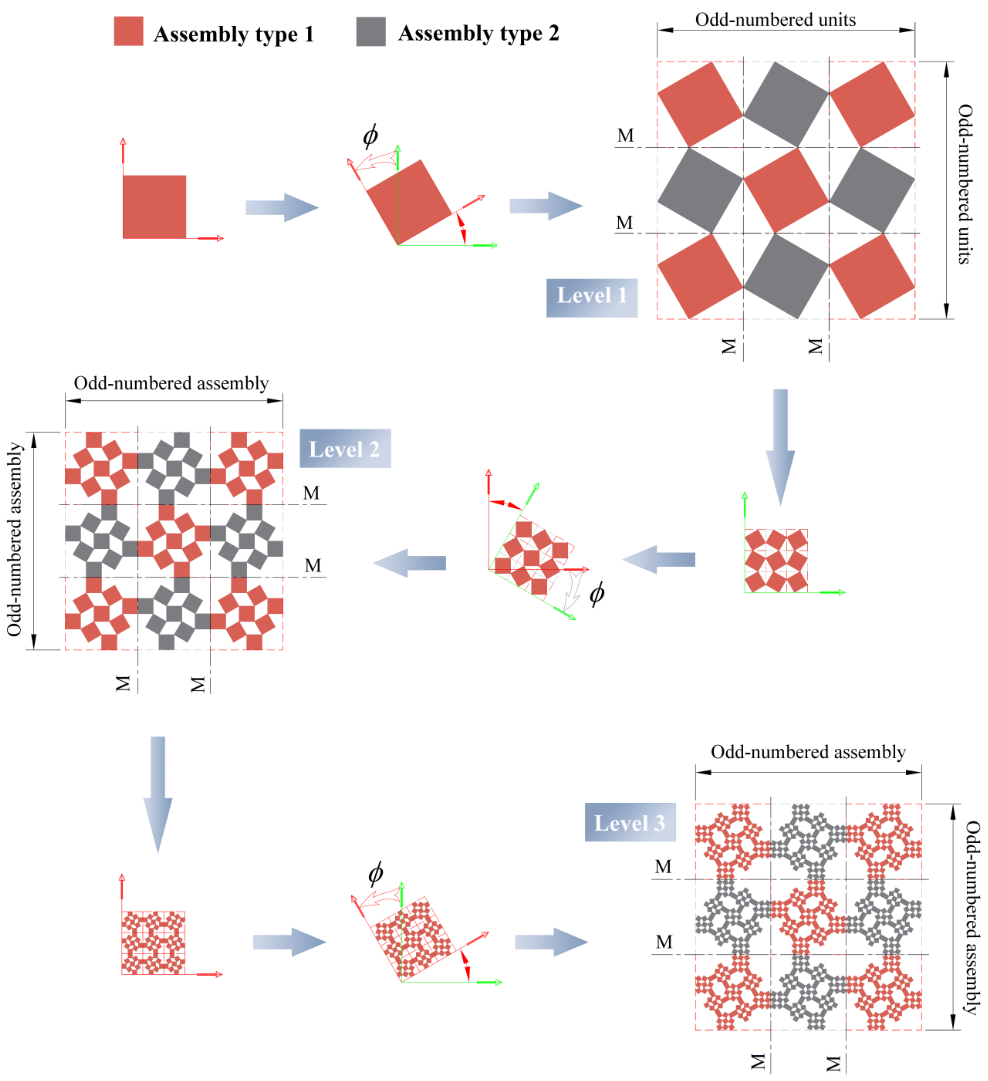

Supplementary Figure 1: *Graphical illustration of the rotate-and-mirror method for constructing a 2D level-3 hierarchical structure. Square rigid unit or assembly from even levels is rotated counter-clockwise firstly and then mirrored (about M planes) to create assembly for constructing the next level hierarchy. Assembly from odd levels is rotated clockwise and mirrored again to create higher level hierarchies.*

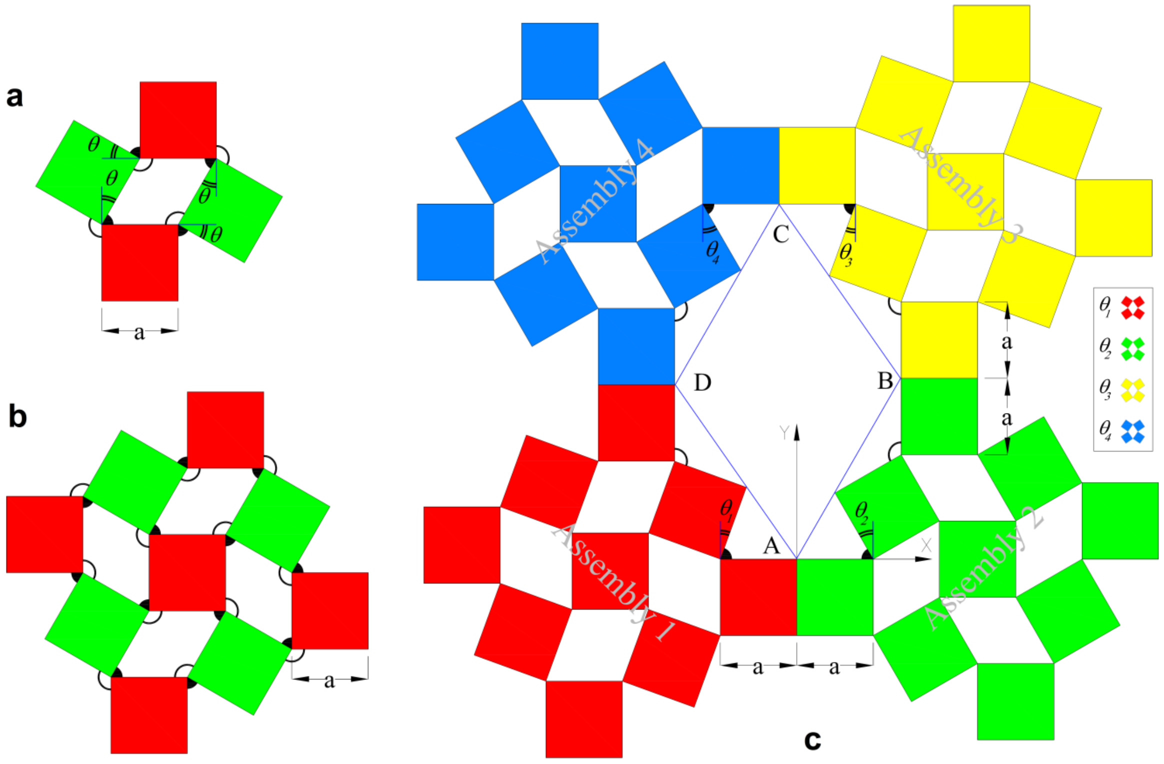

Supplementary Figure 2: *2D hierarchical structures: (a) level-1 structure with a single independent variable θ, (b) level-1 assembly, and (c) level-2 structure with an enclosed void area in the middle.*

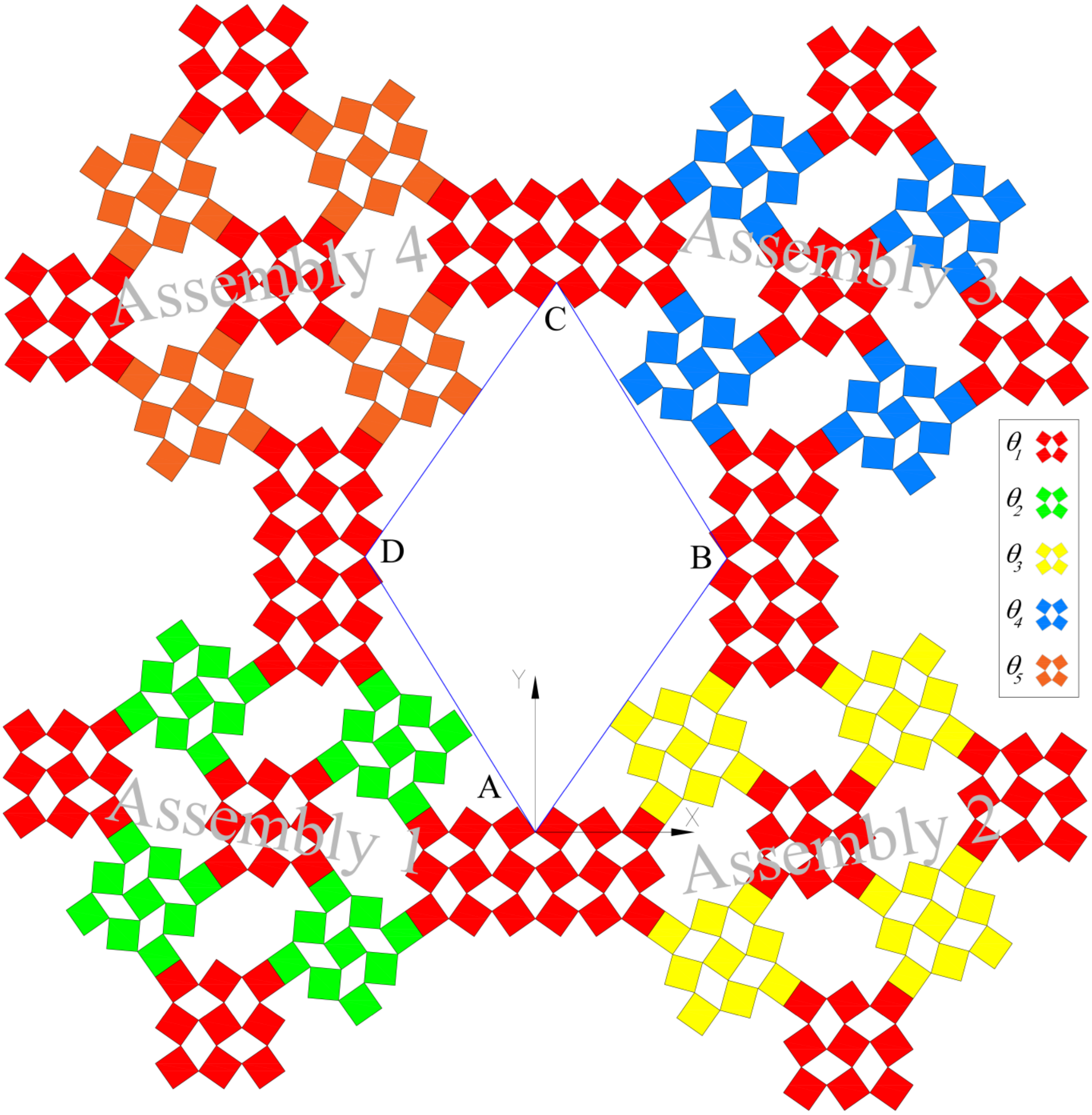

Supplementary Figure 3: *Enclosed void area ABCD of a 2D level-3 hierarchical structure, assuming it has five independent variables from θ_1 to θ_5.*

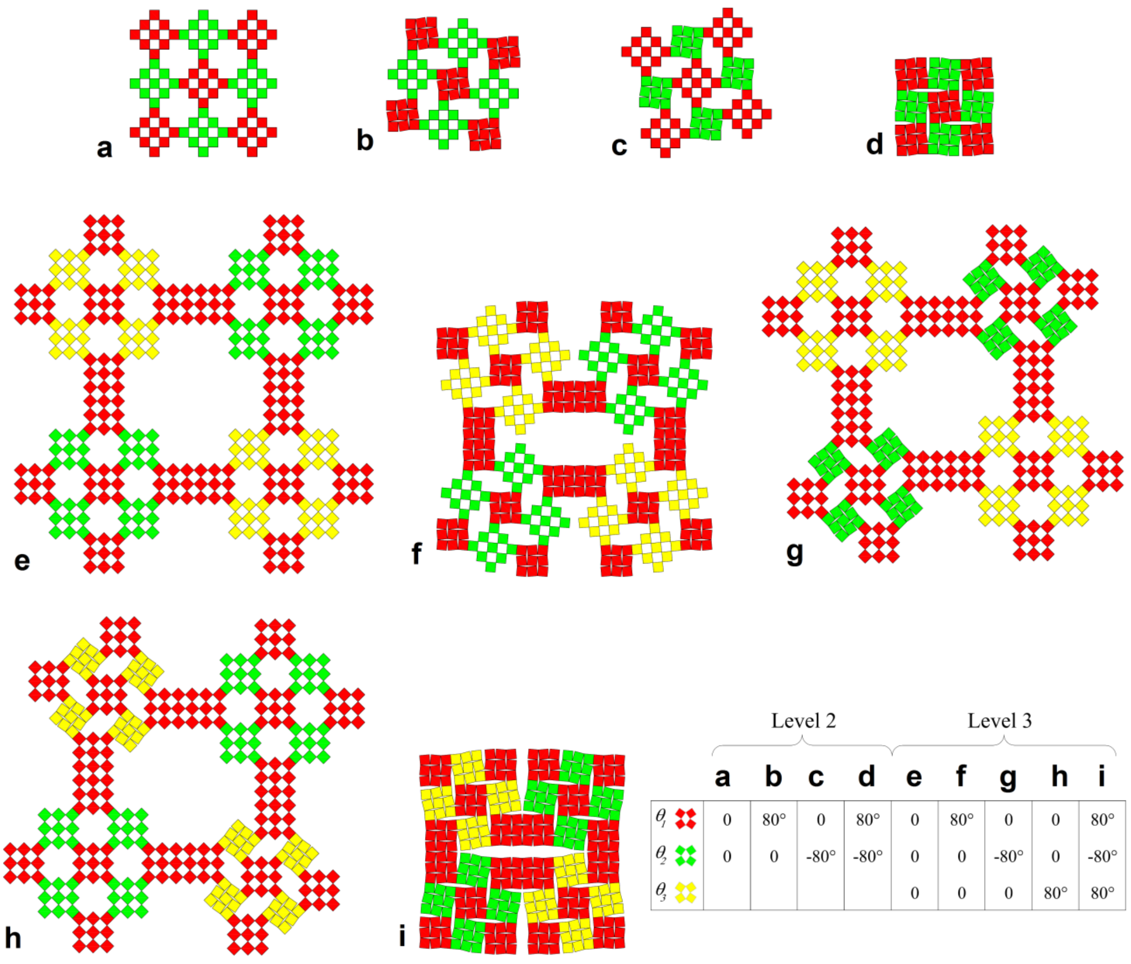

	Level 2				Level 3				
	a	b	c	d	e	f	g	h	i
θ_1	0	80°	0	80°	0	80°	0	0	80°
θ_2	0	0	-80°	-80°	0	0	-80°	0	-80°
θ_3					0	0	0	80°	80°

Supplementary Figure 4: *Level-2 and level-3 hierarchical structures constructed by the rotate-and-mirror method: (a to d), level-2 structures and the deformation patterns when individual degrees are retrained (b, c) and when both degrees of freedom are released (d); (e) to (i), level-3 hierarchical structures when individual degrees are retrained (f to h) and when all three degrees of freedom are released (i).*

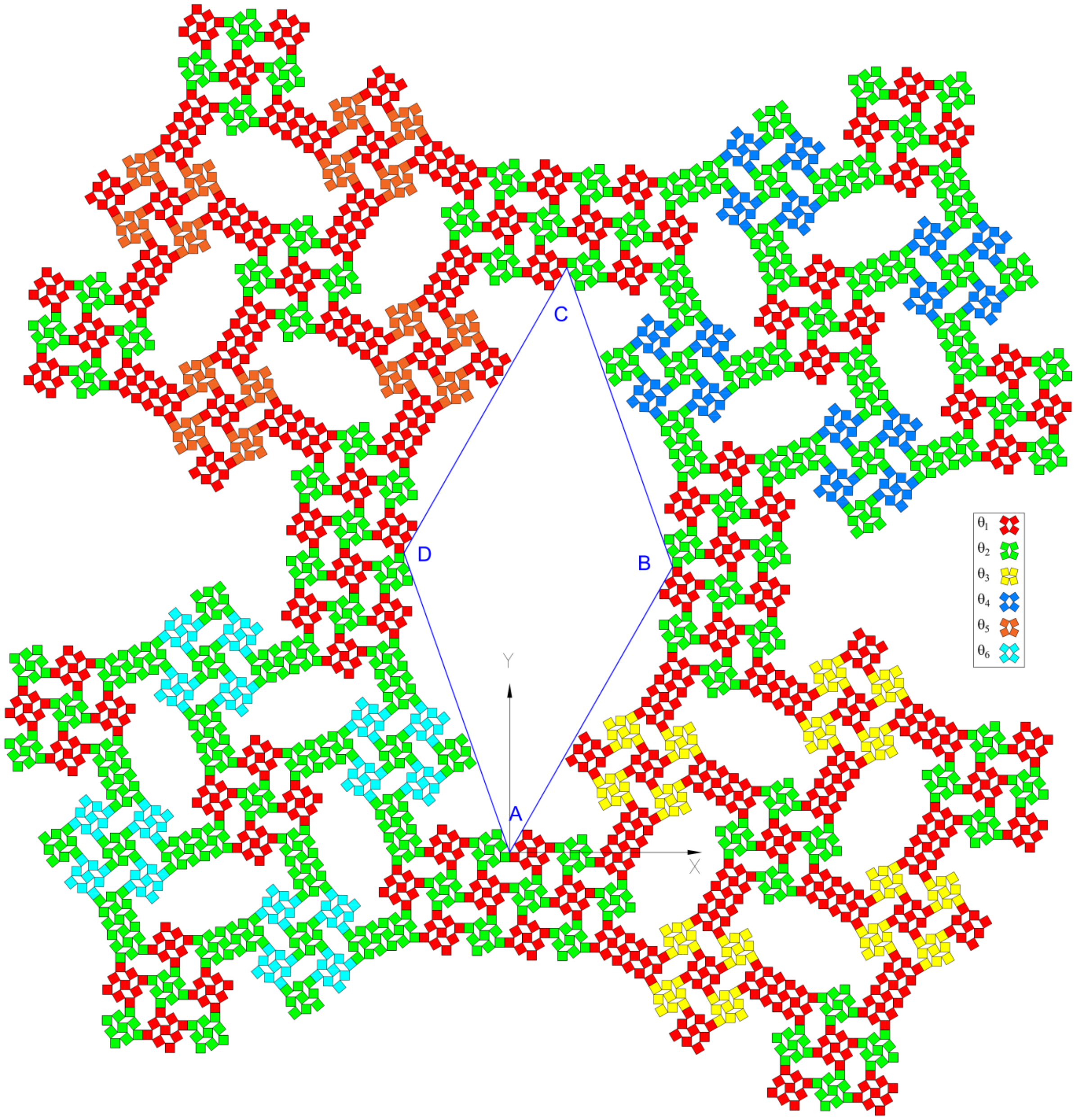

Supplementary Figure 5: *Enclosed void area ABCD of a 2D level-4 hierarchical structure, assuming it has six independent variables from* θ_1 *to* θ_6*.*

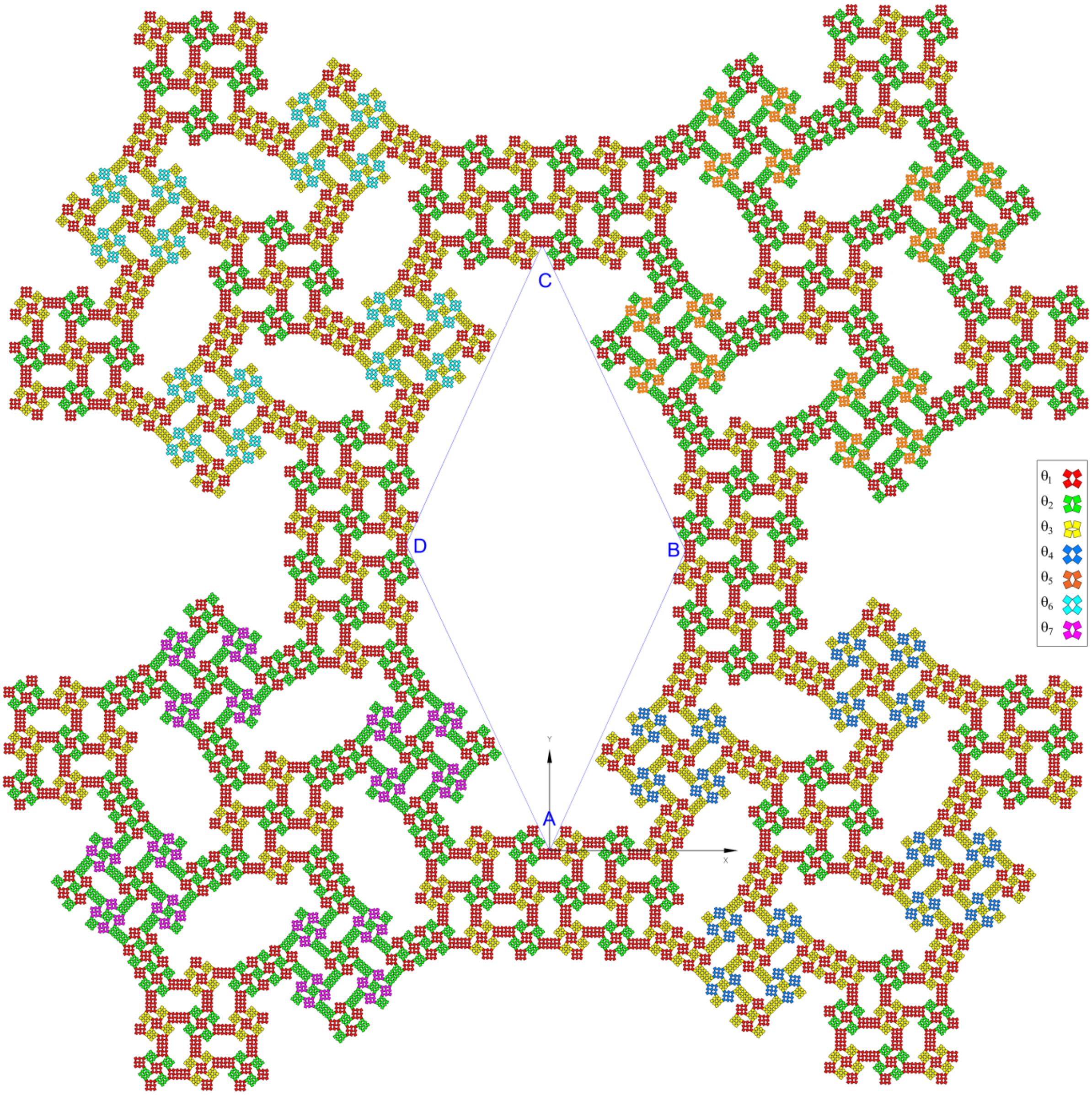

Supplementary Figure 6: *Enclosed void area ABCD of a 2D level-5 hierarchical structure, assuming it has seven independent variables from* θ_1 *to* θ_7.

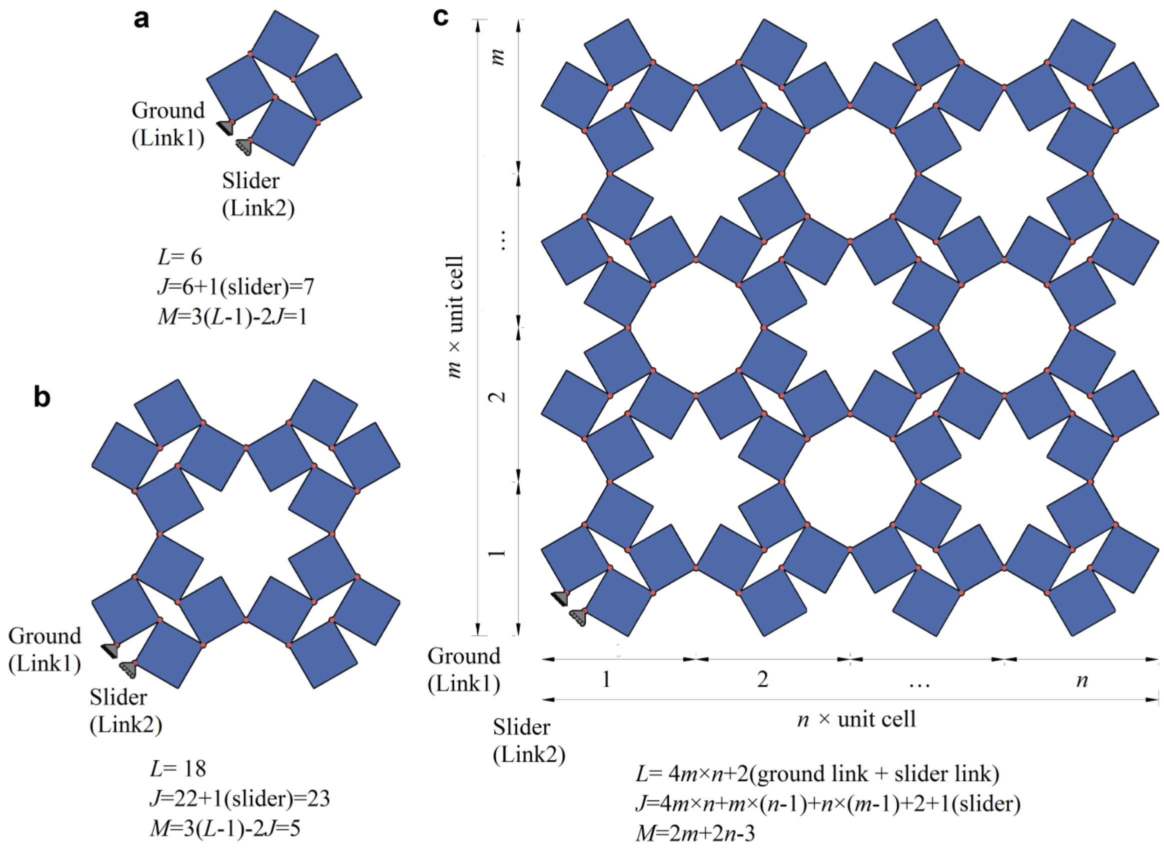

Supplementary Figure 7: *Determining degrees of freedom of level-1 and level-2 fractal cut hierarchies by using Gruebler's equation.*

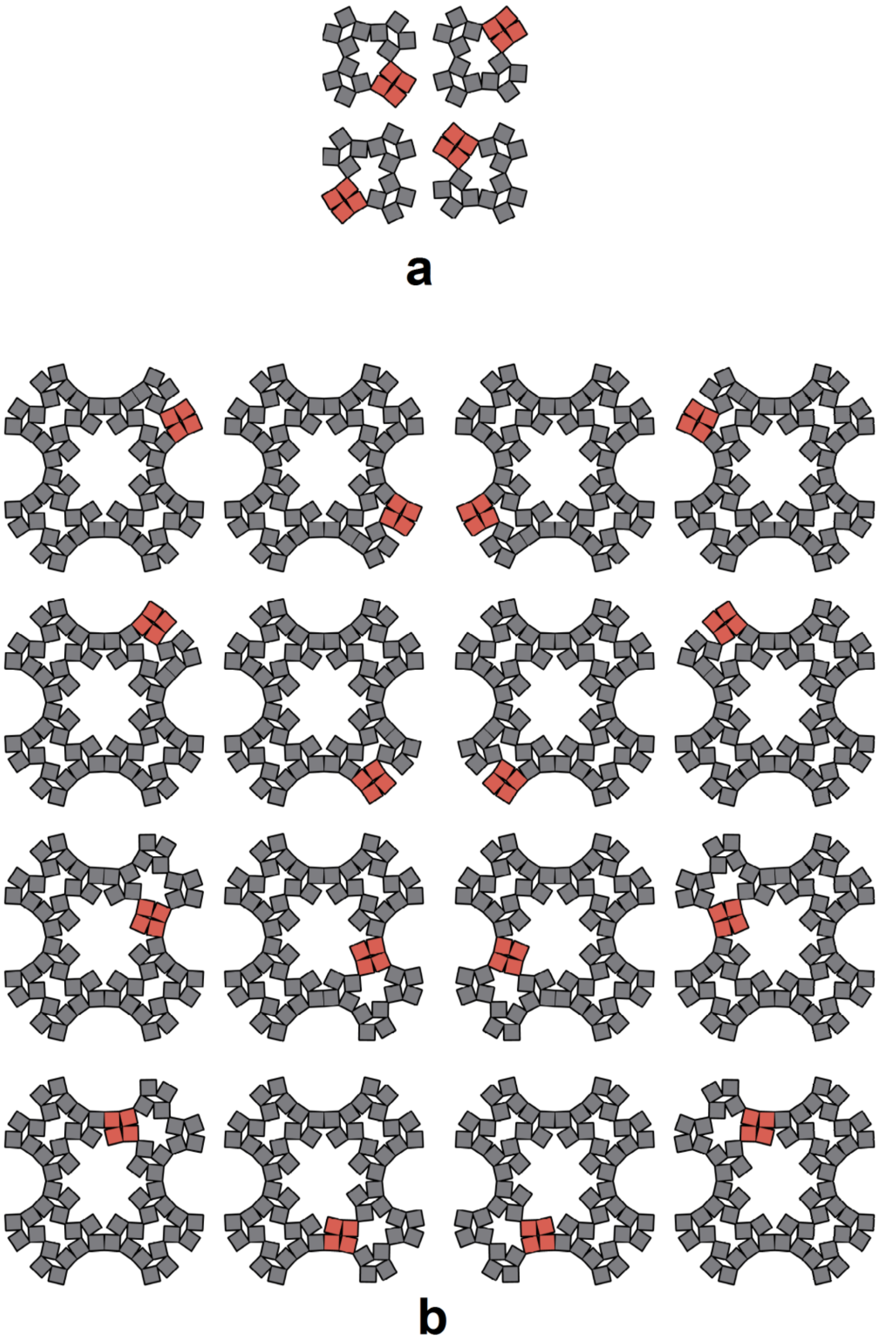

Supplementary Figure 8: *Some of the possible independent variables for level-2 (A) and level-3 (B) hierarchies created by the fractal cut method.*

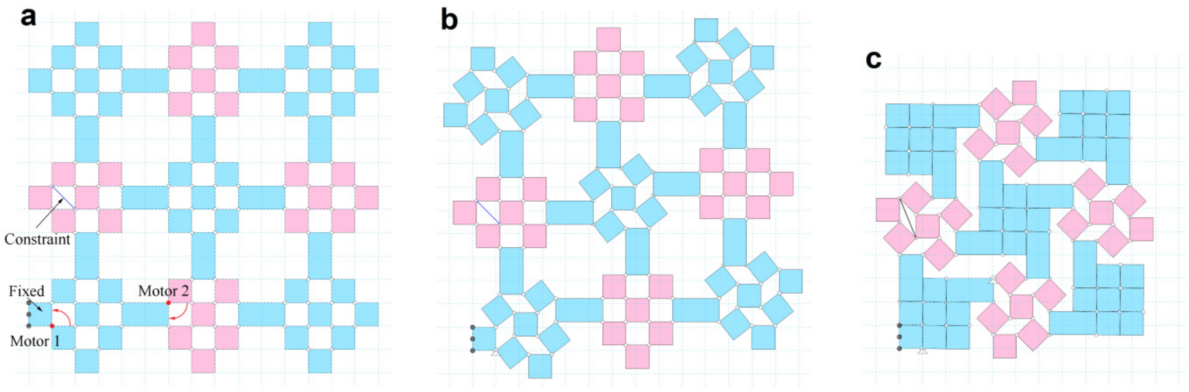

Supplementary Figure 9: *Two-step closing procedure of a level-2 hierarchical structure in Working Model simulation: (A) original structure with the bottom-left vertex unit fixed, (B) displacement constraint activated in step one with motor 1 providing momentum to drive the dependent rotation of the five blue assemblies (assembly type 1), and (C) displacement constraint deactivated in step two with motor 2 providing dependent rotation of the four pink assemblies (assembly type 2).*

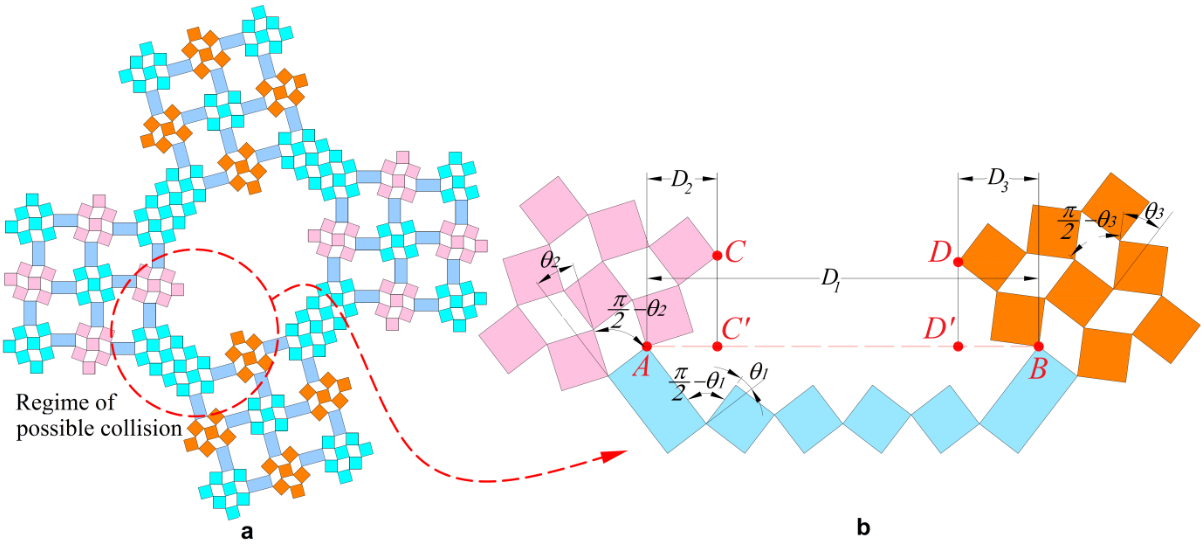

Supplementary Figure 10: *Regimes within a level-3 hierarchical structure that might collide to each other during the closing process: (A) possible regime as highlighted in the dashed circle, (B) an enlarged view for determining the length of segments AB, AC′ and BD′ when rotating the three types of assemblies.*

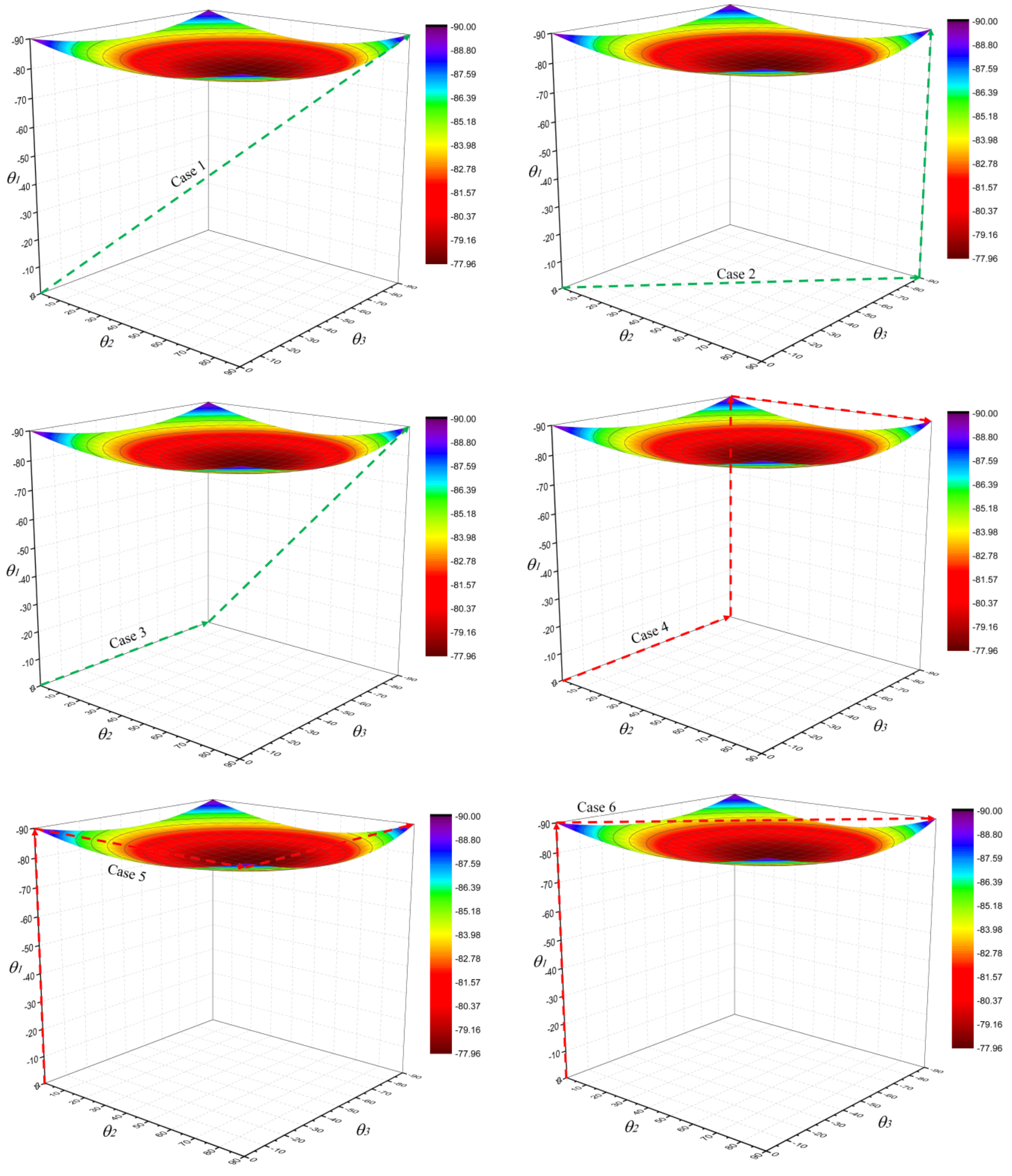

Supplementary Figure 11: *Illustration of the closing routes showing the lower bound of* θ_1*, below which there are no collision occur (Cases 1, 2 and 3) while above which collision occurs (Cases 4, 5 and 6) when the structure closes from fully open state (point A) to fully closed state (point B).*

Supplementary Table 1: *Constructing procedure 3D hierarchical structure level.*

Level-*i* unit/assembly	Rotate: using transformation matrix **T** on odd levels or $\mathbf{T}^{-1}$ on even levels	Mirror: level-(*i*+1) hierarchies
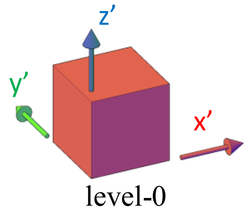 level-0	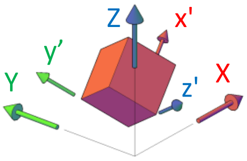	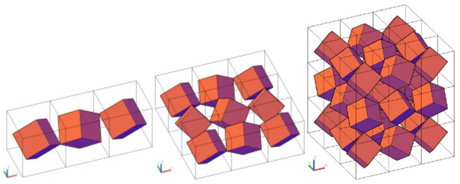 level-1
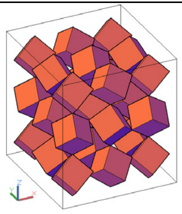 level-1	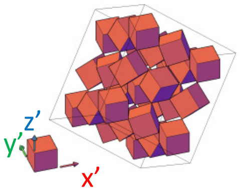	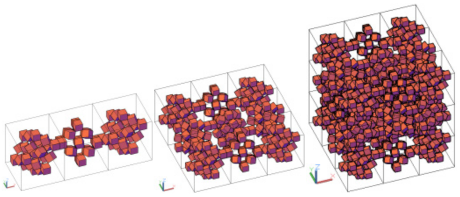 level-2
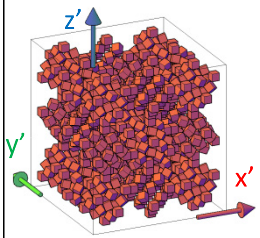 level-2	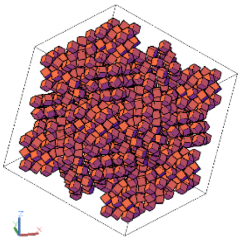	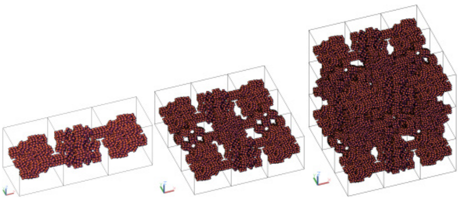 level-3

Supplementary Note 1: Construction of planar hierarchical structures

The rotate-and-mirror method for creating 2D hierarchical structures consists of two steps. A detailed graphical illustration for constructing a level-3 hierarchy is given in Supplementary Fig. 1. Firstly, a square rigid unit (level-0) is rotated *counter-clockwise* about a selected vertex from the original coordinate system (X, Y) to a new system (x', y'), by an angle of ϕ. Then the rotated unit is mirrored about the vertical or horizontal line in (X, Y) system, which passes through one of the four vertices. Continuing the mirror procedure to the rigid unit creates a 3×3 assembly, i.e., level-1 hierarchy. This 3×3 assembly will be treated as an equivalent unit for constructing level-2 hierarchy after being rotated *clockwise* by an angle of ϕ. Continuing the rotate-and-mirror procedure will produce hierarchies of higher levels, as long as the rotational direction is counter-clockwise for assembly/unit from even levels and clockwise for assembly from odd levels. This simple rule guarantees that the assemblies are joined together by connecting adjacent units at the edges of the assemblies by two vertices.

The counter-clockwise rotation from coordinate system (X, Y) to a new system (x', y') can be realized by

$$\begin{bmatrix} X \\ Y \end{bmatrix} = T \begin{bmatrix} x' \\ y' \end{bmatrix}, \text{ when the hierarchy level of the building unit/assembly } N = 0, 2, 4, \ldots \quad \text{[S1]}$$

while the clockwise rotation from (x', y') to (X, Y) can be realized by

$$\begin{bmatrix} x' \\ y' \end{bmatrix} = T^{-1} \begin{bmatrix} X \\ Y \end{bmatrix}, \text{ when the hierarchy level of the building unit/assembly } N = 1, 3, 5, \ldots \quad \text{[S2]}$$

where the transformation matrix T is

$$T = \begin{bmatrix} \cos\phi & -\sin\phi \\ \sin\phi & \cos\phi \end{bmatrix}, \; 0 < \phi \le 90^\circ$$

When ϕ=45°, the structure is in its fully expanded state and $T = \frac{\sqrt{2}}{2}\begin{bmatrix} 1 & -1 \\ 1 & 1 \end{bmatrix}$.

It should be pointed out that the two types of construction assemblies (types 1 and 2) as mentioned in the paper could contain different numbers of units, resulting in a variety of hierarchical structures with different patterns. Theoretically, infinite levels of 2D hierarchies could be achieved through the simple rotate-and-mirror procedure. Practically, however, only limited levels are useful depending on specific applications and manufacturing constraints. A key reason for limited applications of high level hierarchies is the significantly increased degrees of freedom.

Supplementary Note 2: Degrees of freedom of hierarchical structures constructed by the rotate-and-mirror method

The degrees of freedom can be determined by geometrical and mathematical analyses on the void space of the hierarchies. Supplementary Fig. 2 shows void spaces of level-1 and level-2 hierarchies. For level-1 structure, the rotation of the entire structure is governed by a

single variable θ due to geometric constraints as illustrated in Supplementary Fig. 2a. Angles for the level-1 assembly are shown in Supplementary Fig. 2b. The enclosed void area of level-2 structure is surrounded by four sub-level assemblies (Supplementary Fig. 2c). Assuming the rotation of sub-level assemblies are independent of each other, it will have four unknown angles θ_j (j=1 to 4).

Assuming that there are four independent variables θ_j (j=1 to 4) for the level-2 hierarchy in Supplementary Fig. 2c, the relationship between these four variables can be determined by considering the geometric constraints. The void space of the level-2 structure can be simplified to a polygon ABCD, whose horizontal and vertical projections of each edge, Δx and Δy, can be calculated by:

$$\Delta x_{AB} = a(1+\cos\theta_2 - \sin\theta_2),\ \Delta y_{AB} = a(1+\cos\theta_2 + \sin\theta_2);$$

$$\Delta x_{BC} = a(-1-\cos\theta_3 + \sin\theta_3),\ \Delta y_{BC} = a(1+\cos\theta_3 + \sin\theta_3);$$

$$\Delta x_{CD} = a(-1-\cos\theta_4 + \sin\theta_4),\ \Delta y_{CD} = a(-1-\cos\theta_4 - \sin\theta_4);$$

$$\Delta x_{DA} = a(1+\cos\theta_1 - \sin\theta_1),\ \Delta y_{DA} = a(-1-\cos\theta_1 - \sin\theta_1).$$

where a is the size of the rigid unit. Since the polygon ABCD is enclosed, we have $\sum \Delta x = \sum \Delta y = 0$. Hence, $\theta_1=\theta_3$ and $\theta_2=\theta_4$. It shows that the two diagonal assemblies which enclose the void area are dependent on each other. Therefore, level-2 hierarchies constructed by the rotate-and-mirror method have two independent variables, i.e., F_2=2. It should be noted that the degrees of freedom of level-2 hierarchy are independent of the number of assemblies in horizontal and vertical directions, which is fundamentally different from hierarchical structures constructed by the fractal cut method.

For the level-3 hierarchical structure, similar void area can be considered to determine the degrees of freedom, as shown in Supplementary Fig. 3. Assuming that there are five independent variables in the structure, the horizontal and vertical projections of each edge of the polygon ABCD, Δx and Δy, can be calculated by:

$$\Delta x_{AB} = a\left(7\sin(\frac{\pi}{4}-\frac{\theta_1}{2}) + 2\cos(\frac{\pi}{4}-\frac{\theta_1}{2}) + 2\sin(\frac{\pi}{4}-\frac{\theta_1}{2}-\theta_3)\right)$$

$$\Delta x_{BC} = a\left(-7\sin(\frac{\pi}{4}-\frac{\theta_1}{2}) - 2\cos(\frac{\pi}{4}-\frac{\theta_1}{2}) - 2\sin(\frac{\pi}{4}-\frac{\theta_1}{2}-\theta_4)\right)$$

$$\Delta x_{CD} = a\left(-7\sin(\frac{\pi}{4}-\frac{\theta_1}{2}) - 2\cos(\frac{\pi}{4}-\frac{\theta_1}{2}) - 2\sin(\frac{\pi}{4}-\frac{\theta_1}{2}-\theta_5)\right)$$

$$\Delta x_{DA} = a\left(7\sin(\frac{\pi}{4}-\frac{\theta_1}{2}) + 2\cos(\frac{\pi}{4}-\frac{\theta_1}{2}) + 2\sin(\frac{\pi}{4}-\frac{\theta_1}{2}-\theta_2)\right)$$

$$\Delta y_{AB} = a\left(7\cos(\frac{\pi}{4}-\frac{\theta_1}{2}) + 2\sin(\frac{\pi}{4}-\frac{\theta_1}{2}) + 2\cos(\frac{\pi}{4}-\frac{\theta_1}{2}-\theta_3)\right)$$

$$\Delta y_{BC} = a\left(7\cos(\frac{\pi}{4}-\frac{\theta_1}{2}) + 2\sin(\frac{\pi}{4}-\frac{\theta_1}{2}) + 2\cos(\frac{\pi}{4}-\frac{\theta_1}{2}-\theta_4)\right)$$

$$\Delta y_{CD} = a\left(-7\cos(\frac{\pi}{4}-\frac{\theta_1}{2}) - 2\sin(\frac{\pi}{4}-\frac{\theta_1}{2}) - 2\cos(\frac{\pi}{4}-\frac{\theta_1}{2}-\theta_5)\right)$$

$$\Delta y_{DA} = a\left(-7\cos(\frac{\pi}{4} - \frac{\theta_1}{2}) - 2\sin(\frac{\pi}{4} - \frac{\theta_1}{2}) - 2\cos(\frac{\pi}{4} - \frac{\theta_1}{2} - \theta_2) \right)$$

Since the polygon ABCD is enclosed, $\sum \Delta x = \sum \Delta y = 0$. It can be determined that $\theta_2=\theta_4$ and $\theta_3=\theta_5$. Therefore, level-3 hierarchy created by the rotate-and-mirror method has three independent variables, i.e., $F_3=3$. Supplementary Fig. 4 shows the fully expanded structures of level-2 (Supplementary Fig. 4a) and level-3 (Supplementary Fig. 4e) hierarchies as well as various deformation states. Note that rigid units of the same color belong to the same group, and each group would rotate synchronously.

By using similar method, the degrees of freedom for level-4 and level-5 structures can be determined. For a level-*i* hierarchy, we may define *i*+2 characteristic angles θ_j (j=1 to i+2). For example, we may assume that level-4 structure has six independent variables (Supplementary Fig. 5), while level-5 structure has seven (Supplementary Fig. 6). Mathematical calculations prove that both the two hierarchical structures have two variables dependent, respectively. Therefore, $F_4=4$ and $F_5=5$. Generally, the degree of freedom for level-*N* hierarchical structures created by using the rotate-and-mirror method shall follow $F_N=N$.

Supplementary Note 3: Degrees of freedom of hierarchical structures constructed by the fractal cut method

Gruebler's equation (1) is the most commonly used for determining DOF or mobility of a planar structure.

$$M = 3(L-1) - 2J \qquad [S3]$$

where *M* is the mobility or degrees of freedom of a structure, *L* is the number of links or solid elements and *J* is the number of joints. Level-2 fractal cut hierarchy is investigated by using Gruebler's equation.

Supplementary Fig. 7 shows the degrees of freedom for several fractal cut hierarchies. Although level-1 hierarchy has only one degree of freedom, the number of DOF for level-2 hierarchies from the fractal cut method varies, depending on the number of assemblies along the edges, *m* and *n*. In the simplest case, $m=n=2$, the DOF is five. In more general cases, the DOF is equal to $2m+2n+3$. Motion simulations by Working Model reveal the possible independent variables of level-2 and level-3 hierarchies as shown in Supplementary Fig. 8. Different from the hierarchies created by the rotate-and-mirror method, the diagonal assemblies of the fractal cut designs may rotate independently.

Supplementary Note 3: Computational simulation

We apply Working Model in this study to simulate the deformation of 2D hierarchical structures of different levels. The degrees of freedom of level-2 and level-3 hierarchical structures are verified, as well as the deformation patterns. Supplementary Fig. 9 shows the motion of a level-2 hierarchy during a closing process. In this model, five blue assemblies represent the first degree of freedom (type 1 assembly) and four pink assemblies represent the second degree of freedom (type 2 assembly). The simulation is conducted in two steps. In the first step, motor 1 (Supplementary Fig. 9a) applies a clockwise momentum to one the rigid

units in one of the five blue assemblies. In the second step, motor 2 applies a momentum in the opposite direction (clockwise) to one of the rigid units in one of the four pink assemblies. A displacement constraint is imposed in a type 2 assembly during step one, and this constraint is deactivated in step two. One edge of a rigid vertex unit is fixed throughout the simulation. Supplementary Video 1 shows the whole closing procedure of this level-2 hierarchy, clearly demonstrating the synchronized motion of the assemblies of the same type. The motions of the two types of assemblies are independent of each other. Therefore the degrees of freedom for level-2 structure are two, F_2=2.

We have also carried out simulations of hierarchical patterns extracted from the fractal cut method under identical boundary and "loading" conditions. Supplementary Video 2 show four independent degrees of freedom for the level-2 hierarchical structure, i.e. F_2=4. If more rigid units are included in such a level-2 pattern, the degrees of freedom will increase rapidly, while the level-2 structure from the rotate-and-mirror method will always have two degrees of freedom no matter many rigid units are included in the structure.

A level-3 hierarchy from the rotate-and-mirror method containing four level-2 assemblies is simulated in a similar manner using Working Model (Fig. 6a-e). Supplementary Video 3 show the three independent variables in the closing process.

It should be pointed out that rigid units belonging to different assembly types could collide with each other during the closing process. To ensure a successful full closure, the mechanisms of collision are analytically investigated.

The distance between points A, C′, D′ and B can be determined mathematically in the process of rotating different assemblies. In the case of level-3 hierarchical structure shown in Supplementary Fig. 10, there are three independent variables, θ_1, θ_2 and θ_3. To prevent the collision, the length of segments AB, AC′ and BD′, D_1, D_2 and D_3 must satisfy the condition $D_1 \geq D_2 + D_3$ when rotating different types of assemblies. D_1, D_2 and D_3 can be obtained as

$$D_1 = 2\sqrt{2}a(3\cos\frac{\theta_1}{2} - \sin\frac{\theta_1}{2}) \quad \text{[S4a]}$$

$$D_2 = \frac{\sqrt{2}}{2}a\left[(2\cos\theta_2 + 1)\sin\frac{\theta_1}{2} + (2\sin\theta_2 + 1)\cos\frac{\theta_1}{2}\right] \quad \text{[S4b]}$$

$$D_3 = \frac{\sqrt{2}}{2}a\left[(2\cos\theta_3 + 1)\sin\frac{\theta_1}{2} + (-2\sin\theta_3 + 1)\cos\frac{\theta_1}{2}\right] \quad \text{[S4c]}$$

where a is the size of the square rigid unit. Therefore, to prevent collision, we have

$$\theta_1 \geq -2\tan^{-1}\left(\frac{-\sin\theta_2 + \sin\theta_3 + 5}{\cos\theta_2 + \cos\theta_3 + 3}\right) \quad \text{[S5]}$$

Based on Eq. S5, the rotating rigid units in different assemblies would not collide when proper closing procedures are designed. As shown in Supplementary Fig. 11, the curved surface plotted from Eq. S5 defines a lower bound (ceiling) of θ_1, above which collision would occur (Cases 4, 5 and 6). Therefore, to achieve fully closure without collision, one may simply select a route in the diagram from point A (fully open) to point B (fully closed)

without crossing the ceiling. Supplementary Videos 4 and 5 show the closing processes of cases 1 to 6, respectively, which confirm the validity of the collision condition we have discovered.

Supplementary Note 5: Extension to 3D Hierarchies

The rotate-and-mirror method can be easily extended to constructing 3D hierarchical structures with low degrees of freedom and synchronized deformation. Similar to 2D hierarchies, the first step to construct a 3D hierarchical structure is to rotate a cubic rigid unit or a cubic assembly by using a transformation matrix **T** on even levels and $\mathbf{T}^{-1}$ on odd levels,

$$\begin{bmatrix} X \\ Y \\ Z \end{bmatrix} = T \begin{bmatrix} x' \\ y' \\ z' \end{bmatrix}, \text{ and } \begin{bmatrix} x' \\ y' \\ z' \end{bmatrix} = T^{-1} \begin{bmatrix} X \\ Y \\ Z \end{bmatrix} \quad [S6]$$

$$T = \begin{bmatrix} 1+\sin^2(\phi)(\cos(\alpha)-1) & \sin(\phi)cos(\phi)(\cos(\alpha)-1) & -\sin(\phi)\sin(\alpha) \\ \sin(\phi)cos(\phi)(\cos(\alpha)-1) & 1+cos^2(\phi)(\cos(\alpha)-1) & -\cos(\phi)\sin(\alpha) \\ \sin(\phi)\sin(\alpha) & \cos(\phi)\sin(\alpha) & \cos(\alpha) \end{bmatrix}, \quad \begin{matrix} 0 \le \phi \le 90^\circ \\ 0 \le \alpha \le 90^\circ \end{matrix}$$

where ϕ is the angle to rotate the unit about z axis and α is angle to rotate about x axis (columns 2 and 3 of Supplementary Table 1).

We can then mirror the rotated cubic unit/assembly about faces passing through the vertices and perpendicular to the XOY, XOZ and YOZ planes in the original coordinate system. By continuing to mirror the unit/assembly, a hierarchical structure with desired units can be constructed. This simple rotate-and-mirror procedure can be used to create 3D hierarchical structures of any level. Similar to the 2D case, the numbers of units along different edges of the units should be equal and odd. An illustration of the procedure is given in Supplementary Table 1.

Compared to 2D hierarchies, 3D hierarchical structures have higher degrees of freedom. For example, the degrees of freedom of a 3D level-2 hierarchy are 4, in contrast to 2 for its 2D counterpart.

Supplementary References:

1. Angeles, J., Rational Kinematics, Springer-Verlag, New York, pp.99-102 (1988).

Supplementary Video 1. Closing process of a level-2 hierarchy created by the rotate-and-mirror method, showing two degrees of freedom for movement.

Supplementary Video 2. Closing process of a level-2 hierarchy created by the fractal cut method showing five degrees of freedom.

Supplementary Video 3. Closing process of a level-3 hierarchy created by the rotate-and-mirror method showing three degrees of freedom.

Supplementary Video 4. Closure of a level 3 hierarchy along different routes without collision: Cases 1 to 3.

Supplementary Video 5. Closure of a level 3 hierarchy along different routes with collision: Cases 4 to 6.

Supplementary Video 6. Experimental realization of a pin-connected level-2 hierarchical structure, showing two degrees of freedom.
